# Epidemiology of total hip arthroplasty: demographics, comorbidities and outcomes

**DOI:** 10.1186/s42836-022-00156-1

**Published:** 2023-01-03

**Authors:** Ishan Patel, Fong Nham, Leo Zalikha, Mouhanad M. El-Othmani

**Affiliations:** 1 DMC Orthopaedics & Sports Medicine, 3990 John R Street, Detroit, MI 48201 USA; 2 Department of Orthopaedic Surgery, Columbia University Medical Center, 622 W 168th Street, New York, NY 10032 USA

**Keywords:** Hip, Arthroplasty, Demographics, Epidemiology, Outcomes

## Abstract

**Background:**

Primary THA (THA) is a successful procedure for end-stage hip osteoarthritis. In the setting of a failed THA, revision total hip arthroplasty (rTHA) acts as a salvage procedure. This procedure has increased risks, including sepsis, infection, prolonged surgery time, blood loss, and increased length of stay. Increasing focus on understanding of demographics, comorbidities, and inpatient outcomes can lead to better perioperative optimization and post-operative outcomes. This epidemiological registry study aimed to compare the demographics, comorbidity profiles, and outcomes of patients undergoing THA and rTHA.

**Methods:**

A retrospective review of discharge data reported from 2006 to the third quarter of 2015 using the National Inpatient Sample registry was performed. The study included adult patients aged 40 and older who underwent either THA or rTHA. A total of 2,838,742 THA patients and 400,974 rTHA patients were identified.

**Results:**

The primary reimbursement for both THA and rTHA was dispensed by Medicare at 53.51% and 65.36% of cases respectively. Complications arose in 27.32% of THA and 39.46% of rTHA cases. Postoperative anemia was the most common complication in groups (25.20% and 35.69%). Common comorbidities in both groups were hypertension and chronic pulmonary disease. rTHA indications included dislocation/instability (21.85%) followed by mechanical loosening (19.74%), other mechanical complications (17.38%), and infection (15.10%).

**Conclusion:**

Our data demonstrated a 69.50% increase in patients receiving THA and a 28.50% increase in rTHA from the years 2006 to 2014. The data demonstrated 27.32% and 39.46% complication rate with THA and rTHA, with postoperative anemia as the most common cause. Common comorbidities were hypertension and chronic pulmonary disease. Future analyses into preoperative optimizations, such as prior consultation with medical specialists or improved primary hip protocol, should be considered to prevent/reduce postoperative complications amongst a progressive expansion in patients receiving both THA and rTHA.

## Background

Primary total hip arthroplasty (THA) constitutes the standard of care for treatment of end-stage hip osteoarthritis and provides pain relief and improved joint function [1]. The prevalence of THA in the United States among adults fifty years of age or older was estimated to be 2.34% in 2010. It is predicted that demand and volume of this procedure will increase in coming years due to higher demand for improved mobility and quality of life in an aging population [2]. While THA is a successful procedure and among the top five most common and fastest-growing procedures in the United States, its failure, for reasons such as instability, infection, loosening, and implant failure or wear, poses a significant burden to patients and the national healthcare system [3]. In the setting of a failed THA, revision total hip arthroplasty (rTHA) is a salvage procedure with increased surgical risks, including sepsis, prosthetic joint infection, prolonged surgical time and exposure to anesthesia, increased blood loss, cost of care, and hospital length of stay (LOS) [4]. As the incidence of THA increases, a 43% to 70% increase in frequency of rTHA from 2014 to 2030 has been similarly projected [4].

Over the last decade, with the conceptualization of value-based care, healthcare systems witnessed a shift in focus towards improving quality while simultaneously minimizing cost of delivered care. Such efforts have gained substantial traction among high-volume and high-impact procedures, such as THA. The successful, consistent, and reproducible achievement of these targets requires implementation of evidence-based best practices through standardized care pathways. Featherall et al. demonstrated that an implemented standardized protocol of preoperative, intraoperative and postoperative care standards could effectively reduce LOS with increasing home discharge [5].

While this approach has been efficient in improving various postoperative outcomes, there remains considerable room for improvement that can be guided by more advanced perioperative optimization protocols that target the different medical and social comorbidities commonly found among arthroplasty recipients. While previous epidemiological studies focused on demographics and outcomes of THA recipients, there remains a lack of an in-depth understanding of a comprehensive list of the most common medical comorbidities found in THA and rTHA recipients.

As such, the aim of this study was to provide a better understanding of the demographics, comorbidity profile, and in-hospital outcomes of patients undergoing THA and rTHA. Further, the type of revision and the indication for rTHA performed in the United States from the year 2006 to the third quarter of 2015 were reported. In reporting these statistics, the authors aimed to provide a baseline structural understanding of common demographic and medical characteristics of THA and rTHA recipients, which might guide perioperative protocol development and improvement.

## Methods

A retrospective analysis of discharge data from 2006 to the third quarter of 2015 using the NIS registry was undertaken. This database accounts for roughly 20% of inpatient stays across the United States, and includes information such as demographics, comorbidity profiles, in-hospital outcomes, as well as the type and reason for rTHA. The International Classification of Disease, Ninth Revision, Clinical Modification (ICD-9-CM) was used for procedure and diagnosis codes within the NIS during the study period. As the NIS transitioned to ICD-10 coding in the fourth quarter of 2015 we elected to exclude that quarter to maintain homogeneity of the implemented methodology. The inclusion criteria for this study were defined as patients aged at least 40 years who underwent either THA or rTHA. The age of at least 40 years was chosen to include a cohort representative of the elective joint replacement population that would be typically encountered in practice. These patients were identified with ICD-9 code 81.51 (total hip replacement) or 81.53 (revision of total hip replacement). Reasons for revision were also included with the use of ICD-9 codes: 996.42 (dislocation/instability), 996.41 (mechanical loosening), 996.66 (infection), 996.43 (implant failure), 996.45 (periprosthetic osteolysis), 996.44 (periprosthetic fracture), 996.46 (bearing surface wear), 996.47 (other mechanical problems), and 996.49 (other mechanical complications). We elected to combine “other mechanical problems” and “other mechanical complications” into one category in the results. The type of revision was also included with ICD-9 codes: 00.70 (all components), 00.71 (acetabular component), 00.72 (femoral component), 00.73 (acetabular liner and/or femoral head only), 80.05 (arthrotomy for removal of prosthesis), and 81.53 (other not otherwise specified). The variables assessed included demographics, in-hospital outcomes, Elixhauser medical comorbities profile, reason for rTHA, and type of rTHA.

Demographic data collected includes total number of discharges (corresponding to the number of cases), patient age, sex, primary payor, race, calendar year of discharge, hospital bed size, location/teaching status, hospital geographic location, as well as elective or non-elective nature of admission for rTHA.

In-hospital outcome data collected includes patient complications during the inpatient stay. This dataset includes a variable, any complication, as a measure referring to any cardiac, respiratory, peripheral vascular disease (PVD), hematoma/seroma, wound dehiscence, postoperative infection, gastrointestinal complication, genitourinary complication, deep vein thrombosis, pulmonary embolism or postoperative anemia complication. In addition, this data set does include the average cost and length of stay for both primary and revision total hip arthroplasty.

A comorbidity profile was created using the Elixhauser comorbidity index. This index is used frequently in database studies to evaluate patient comorbidities. The index allows for better controlling of potential confounding effects of preexisting diseases [6]. Although this study did not aim to directly compare any variables, presenting the entire Elixhauser comorbidity profile allows for a more comprehensive understanding of the major medical comorbidities of this patient population.

## Results

### Demographic data

**Fig. 1:**
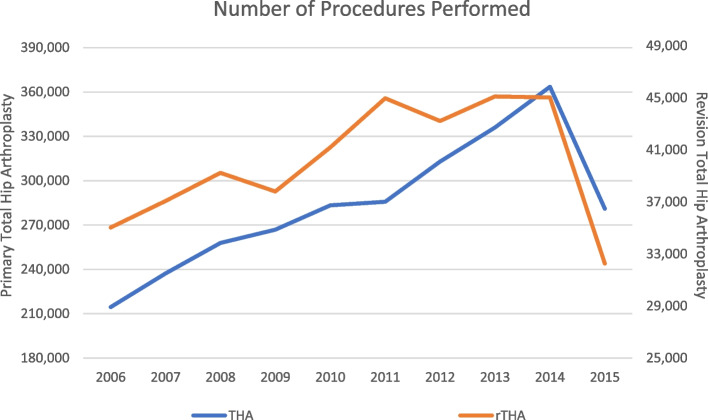
Number of procedures performed. This figure displays the amount of patient undergoing THA and rTHA by year

A total of 2,838,742 THA patients and 400,974 rTHA patients were included in this study. Average age in THA and rTHA groups was 65.98 and 68.54 years, respectively. The major primary payor for both THA and rTHA was Medicare, at 53.51% and 65.36% of cases, respectively, followed by private payor. In terms of race, non-Hispanic whites were by far the majority recipients of THA and rTHA, at 74.80% and 74.40% of discharges, respectively. In terms of number of discharges per year, which can be translated to number of procedures performed per year, according to our results there has been a consistent increase every year between 2006 and 2014 (Fig. 1). Of note, the procedure volume for the year 2015 showed a slight decrease in cases in both cohorts, which can be explained by the exclusion of the data from the 4th quarter from that year as per the study design. The demographic variables of the study population are detailed in Table 1.

**Table 1:** Total hip arthroplasty demographics

	THA	rTHA
	Discharges (***n*** = 2,838,742)	Discharges (***n*** = 400,974)
Age of Patient (Years)		
Mean (Standard Error)	65.98 (0.05)	68.54 (0.08)
Biological Sex of Patient		
Male	1,239,107 (43.65%)	172,950 (43.14%)
Female	1,599,635 (56.35%)	228,024 (56.87%)
Expected Primary Payor		
Medicare	1,519,119 (53.51%)	262,084 (65.36%)
Medicaid	95,921 (3.38%)	15,860 (3.96%)
Private	1,124,965 (39.63%)	108,030 (26.94%)
Self-Pay	20,617 (0.73%)	2,824 (0.70%)
No Charge	3,707 (0.13%)	480 (0.12%)
Other Insurance	74,412 (2.62%)	11,697 (2.92%)
Race of Patient		
Non-Hispanic White	2,123,402 (74.80%)	298,324 (74.40%)
Non-Hispanic Black	171,191 (6.03%)	22,680 (5.66%)
Hispanic	76,260 (2.69%)	12,396 (3.09%)
Other Race	467,889 (16.48%)	67,574 (16.85%)
Calendar Year of Discharge		
2006	214,451 (7.55%)	35,034 (8.74%)
2007	237,237 (8.36%)	37,075 (9.25%)
2008	257,853 (9.08%)	39,243 (9.79%)
2009	266,904 (9.40%)	37,808 (9.43%)
2010	283,275 (9.98%)	41,194 (10.27%)
2011	285,683 (10.06%)	44,980 (11.22%)
2012	312,910 (11.02%)	43,230 (10.78%)
2013	335,900 (11.83%)	45,110 (11.25%)
2014	363,575 (12.81%)	45,030 (11.23%)
2015	281,000 (9.90%)	32,270 (8.05%)
Bedsize of Hospital		
Small	542,124 (19.10%)	65,044 (16.22%)
Medium	740,181 (26.07%)	95,687 (23.86%)
Large	1,547,591 (54.52%)	238,109 (59.38%)
Unknown	8,846 (0.31%)	2,134 (0.53%)
Location/Teaching Status of Hospital		
Rural	277,772 (9.79%)	28,180 (7.03%)
Urban Nonteaching	1,153,501 (40.63%)	143,218 (35.72%)
Urban Teaching	1,398,623 (49.27%)	227,442 (56.72%)
Unknown	8,846 (0.31%)	2,134 (0.53%)
Region of Hospital		
Northeast	566,838 (19.97%)	72,122 (17.99%)
Midwest	746,641 (26.30%)	98,410 (24.54%)
South	939,107 (33.08%)	144,753 (36.10%)
West	586,156 (20.65%)	85,688 (21.37%)
Elective Admission		
Non-Elective	n/a	114,244 (28.49%)
Elective	n/a	285,841 (71.29%)
Unknown	n/a	888 (0.22%)

A comprehensive list of demographic data collected from the NIS database of patients undergoing THA and rTHA from 2006 to the third quarter of 2015

### Elixhauser comorbidity profile

There was a slight variation in the incidence of different comorbidities between the THA and rTHA groups. Hypertension appeared to be the most common comorbidity in both THA and rTHA cohorts, at 60.46% and 59.74%, respectively. The five most prevalent comorbidities in the THA group were hypertension (60.46%), obesity (15.11%), chronic pulmonary disease (14.37%), hypothyroidism (13.68%), and uncomplicated diabetes (13.67%). The five most prevalent comorbidities in the rTHA group included hypertension (59.74%), deficiency anemia (17.51%), chronic pulmonary disease (16.92%), fluid/electrolyte disorders (15.74%), and hypothyroidism (14.81%). The complete Elixhauser comorbidity profile for both THA and rTHA is summarized in Table 2.

**Table 2:** Elixhauser comorbidity profiles

THA		rTHA	
	Discharges (***n*** = 2,838,742)		Discharges (***n*** = 400,974)
Hypertension	1,716,272 (60.46%)	Hypertension	239,561 (59.74%)
Obesity	428,814 (15.11%)	Deficiency Anemias	70,229 (17.51%)
Chronic Pulmonary Disease	407,858 (14.37%)	Chronic Pulmonary Disease	67,859 (16.92%)
Hypothyroidism	388,394 (13.68%)	Fluid and Electrolyte Disorders	63,133 (15.74%)
Diabetes (Uncomplicated)	388,170 (13.67%)	Hypothyroidism	59,399 (14.81%)
Deficiency Anemias	387,928 (13.67%)	Obesity	59,202 (14.76%)
Depression	318,317 (11.21%)	Depression	57,911 (14.44%)
Fluid and Electrolyte Disorder	261,545 (9.21%)	Uncomplicated Diabetes	54,259 (13.53%)
Renal Failure	119,673 (4.22%)	Other Neurological Disorders	28,123 (7.01%)
Valvular Heart Disease	113,545 (4.00%)	Renal Failure	27,955 (6.97%)
Rheumatoid Arthritis/Collagen Vascular Disease	108,372 (3.82%)	Rheumatoid Arthritis/Collagen Vascular Disease	27,602 (6.88%)
Other Neurological Disorders	105,927 (3.73%)	Congestive Heart Failure	24,152 (6.02%)
Congestive Heart Failure	80,299 (2.83%)	Valvular Disease	20,709 (5.17%)
Peripheral Vascular Disorders	68,802 (2.42%)	Coagulopathy	16,292 (4.06%)
Coagulopathy	64,920 (2.29%)	Peripheral Vascular Disorders	13,677 (3.41%)
Psychoses	53,880 (1.90%)	Psychoses	12,344 (3.08%)
Chronic Blood Loss Anemias	50,668 (1.79%)	Weight Loss	12,123 (3.02%)
Alcohol Abuse	48,069 (1.69%)	Alcohol Abuse	9,816 (2.45%)
Diabetes (Complicated)	36,013 (1.27%)	Chronic Blood Loss Anemias	9,295 (2.32%)
Liver Disease	30,597 (1.08%)	Liver Disease	8,051 (2.01%)
Pulmonary Circulation Disorders	24,838 (0.88%)	Complicated Diabetes	6,718 (1.68%)
Drug Abuse	20,699 (0.73%)	Pulmonary Circulation Disorders	6,716 (1.68%)
Weight Loss	16,756 (0.59%)	Drug Abuse	5,708 (1.42%)
Solid Tumor without Metastasis	16,463 (0.58%)	Paralysis	3,848 (0.96%)
Lymphoma	10,662 (0.38%)	Solid Tumor without Metastasis	3,028 (0.76%)
Paralysis	10,199 (0.36%)	Lymphoma	2,261 (0.56%)
Metastatic Cancer	8,225 (0.29%)	Metastatic Cancer	2,240 (0.56%)
Acquired Immune Deficiency Syndrome (AIDS)	3,644 (0.13%)	Acquired Immune Deficiency Syndrome (AIDS)	640 (0.16%)
Peptic Ulcer Disease Excluding Bleeding	494 (0.02%)	Peptic Ulcer Disease Excluding Bleeding	96 (0.02%)

Elixhauser comorbidity profile collected from the NIS database of patients undergoing THA and rTHA from 2006 to the third quarter of 2015

### In-hospital outcomes

The outcomes collected from the NIS database aimed to highlight the complications during the in-hospital stay of patients undergoing pTHA and rTHA. The rate of sustaining any complication was 27.32% for THA and 39.46% for rTHA. In terms of specific complications, the most common complication for both pTHA and rTHA was postoperative anemia, at 25.72% and 35.69%, respectively. In terms of economic postoperative outcomes, THA had a total cost of $53,324 with an average length of stay (LOS) of 3.30 days, while rTHA had a total cost of $75,037 with an average LOS of 5.37 days. Table 3 summarizes all in-hospital outcomes collected for both cohorts.

**Table 3:** Total hip arthroplasty in hospital outcomes

THA		rTHA	
	Discharges (***n*** = 2,838,742)		Discharges (***n*** = 400,974)
Any Complications	775,599 (27.32%)	Any Complication	158,235 (39.46%)
Postoperative Anemia	730,134 (25.72%)	Postoperative Anemia	143,120 (35.69%)
Hematoma/Seroma	22,521 (0.79%)	Hematoma/Seroma	11,539 (2.88%)
Cardiac Complication	19,042 (0.67%)	Postoperative Infection	4,337 (1.11%)
Genitourinary Complication	16,046 (0.57%)	Wound Dehiscence	4,194 (1.05%)
Gastrointestinal Complication	9,198 (0.32%)	Cardiac Complication	3,806 (0.95%)
Deep Vein Thrombosis	5,752 (0.20%)	Died During Hospitalization	3,127 (0.78%)
Pulmonary Embolism	5,348 (0.16%)	Deep Vein Thrombosis	3,034 (0.76%)
Respiratory Complication	4,556 (0.16%)	Genitourinary Complication	2,358 (0.59%)
Died During Hospitalization	4,234 (0.15%)	Respiratory Complication	1,870 (0.47%)
Postoperative Infection	3,246 (0.11%)	Pulmonary Embolism	1,589 (0.40%)
Wound Dehiscence	1,903 (0.07%)	Gastrointestinal Complication	1,511 (0.38%)
Peripheral Vascular Disease Complication	1,242 (0.04%)	Peripheral Vascular Disease Complication	369 (0.09%)
Total Charges, $, (Standard Error)	$53,324 ($478)	Total Charges, $, (Standard Error)	$75,037 ($901)
Length of Stay, Days, (Standard Error)	3.30 (0.01)	Length of Stay, Days, (Standard Error)	5.37 (0.04)

In hospital outcomes collected from the NIS database of patients undergoing THA and rTHA from 2006 to the third quarter of 2015

### Reason/type of rTHA

In the study time period, dislocation and instability (21.85%) appeared to be the most common reason for rTHA. This was followed by mechanical loosening (19.74%), other mechanical complications (17.38%), and then infection (15.10%). In terms of type of revision, the majority of patients underwent revision of all components (42.33%). The femoral component (15.66%) appeared to be revised more often than the acetabular component (14.36%). The findings for reason and type of rTHA are summarized in Tables 4 and 5, respectively.

**Table 4:** Reason for revision total hip arthroplasty

	Discharges (***n*** = 400,974)
Dislocation/Instability	87,605 (21.85%)
Mechanical Loosening	79,156 (19.74%)
Other Mechanical Complications	69,684 (17.38%)
Infection	60,562 (15.10%)
Periprosthetic Osteolysis	27,668 (6.90%)
Periprosthetic Fracture	26,332 (6.57%)
Implant Failure	23,103 (5.76%)
Bearing Surface Wear	22,306 (5.56%)

Listed reason for patients undergoing rTHA collected from the NIS database of patients undergoing THA and rTHA from 2006 to the third quarter of 2015

**Table 5:** Type of revision total hip arthroplasty

	Discharges (***n*** = 400,974)
All Components	169,743 (42.33%)
Femoral Component	62,808 (15.66%)
Acetabular Liner and/or Femoral Head Only	57,875 (14.43%)
Acetabular Component	57,589 (14.36%)
Arthrotomy for Removal of Prosthesis	36,422 (9.08%)
Other, Not Otherwise Specified	20,592 (5.14%)

Listed types of rTHA collected from the NIS database of patients undergoing THA and rTHA from 2006 to the third quarter of 2015

## Discussion

The rates of THA are projected to increase between 43% and 70% from 2014 to 2030, with a corresponding proportional increase in rTHA [4]. Our analysis of 2006 to 2014 data demonstrated a 69.50% increase in patients receiving THA and a 28.50% increase in rTHA. 2015 data were not included in these calculations as they do not contain data from the full year. The increase in patients receiving these procedures may be indicative of expanding indications and increasing demand for THA, potentially driven by an aging population, higher rates of diagnosis and treatment of osteoarthritis, increased demand for improved quality of life [2], and generational improvements in implant design and longevity. As the volume of both index and revision procedures increases yearly, and as reimbursement systems shift towards value-based models, a heightened focus has been placed on resource utilization and quality of care delivery. In the setting of THA, this transition translates into a focus on limiting wasteful resource consumption while simultaneously improving perioperative outcomes. A failed THA results in a rTHA performed in an inpatient setting, with a reported average cost of $75,037 per procedure, totaling over $30,000,000,000 from 2006–2015. In order to decrease this revision burden, there is a need for continuously improving the technical aspects associated with the procedure in addition to perioperative medical and social optimization of THA recipients. Such optimization efforts would be substantially challenging without an in-depth understanding of the major demographic and medical comorbidities of this patient population.

While primary THA is considered one of the most successful procedures in medicine, its failure and subsequent revisions pose a significant negative impact on patient quality of life and the healthcare system. In this study, an increase in the rate of rTHA was noted between 2006 and 2015, with dislocation constituting the most common reason for revision during that time period. Previously reported literature also reported similar findings for reasons for rTHA [7, 8]. Ulrich et al. reported that in a cohort of 237 rTHA, 50% of patients had the revision surgery within five years of the index procedure. Furthermore, most revisions were performed for instability (33%) and infection (24%) [7]. The most crucial independent variable which may predispose patients to dislocation is implant position, specifically positioning of the acetabular shell [9, 10]. Robotic assisted THA, which helps with implant positioning, has been shown to help reduce dislocation rates compared to conventional THA. Shaw et al. found, in their cohort of 2247 patients, that the dislocation rate 0.60% with robotic assisted THA *vs.* 2.50% in the conventional THA cohort [11]. In addition to technical factors, patient characteristics and comorbidities also play a role in dislocation rates. Obesity, dementia, depression, Parkinson’s disease, chronic lung disease and inflammatory arthritis, amongst other factors, serve as independent risk factors to dislocation following THA [12, 13]. Preoperative optimization of these independent risk factors as well as taking care intraoperatively to ensure appropriate implant position may serve to decrease overall need for rTHA.

There are currently about 46 million geriatric adult patients living in the United States. This number is expected to grow to about 90 million geriatric adults based on a 2050 projection estimate [14]. In the years between 2020 and 2030 alone, the projection estimates another 18 million geriatrics adults [14]. Our study data revealed that the average age of patients undergoing pTHA was 65.98 and 68.54 for rTHA. As the population ages and “baby boomers” reach geriatric age, and the reported average age of THA is about 65 years, there is a projected significant increase in the amount of THA and corresponding increase in rTHA procedures. Increasing age would also account for an increased amount of comorbidities which would further impact rates of complications in hip arthroplasty patients.

With regards to other demographic variables, the findings of this study were in line with previously reported literature in terms of average age, gender distribution, and primary payor type, among other variables [15]. In terms of hospital location, we noted a higher rate of procedures performed in the urban setting, with a total of 89.90% for THA and 92.44% for rTHA. More interestingly, teaching institutions constituted a notably different rate between the procedures, with 56.72% for rTHA compared to a 49.27% for THA. While referral patterns of complex revisions for reasons such as lack of expertise or resources at smaller non-teaching centers may in part explain these differences, there are likely also financial incentives at play [16]. Previous literature has demonstrated that contemporary reimbursement models fail to adequately compensate for the additional resource utilization and costs that rTHA requires relative to THA [17, 18]. As such, non-teaching institutions may be incentivized to “cherry pick” primary procedures while “lemon dropping” the more complex and less financially rewarding revision procedures, consequently offloading the additional revision burden to teaching institutions [17, 19, 20]. Such financial disincentives have the potential to lead to access to care issues, and our findings support the idea that the Current Procedural Terminology (CPT) coding system and RVU allocation for revision procedures should be reexamined to better align incentives [21].

This study reported on all comorbidities that constitute the Elixhauser comorbidity index, which has been extensively utilized in epidemiological studies and proven to be a superior tool for outcome prediction at the population level [6, 22, 23]. Understanding the medical comorbidity profile of THA and rTHA recipients might allow for improved preoperative optimization protocols and for development of appropriate risk stratification models that can subsequently guide establishment of fairer reimbursement models. A study by Dlott et al. reported reduced LOS and less subsequent emergency department visits among patients who underwent preoperative optimization [24]. While arthroplasty literature assessing correlation of various medical comorbidities with postoperative outcomes is quite extensive, a complete delineation of the impact of major comorbidities and their interaction on postoperative outcomes remains lacking. The data noted the most common comorbidities in both THA and rTHA groups to be hypertension, obesity, chronic pulmonary disease, hypothyroidism, uncomplicated diabetes, deficiency anemia, fluid/electrolyte imbalance and depression.

Obesity, one of the most studied comorbidities in arthroplasty, was found to be the second highest comorbidity in the THA group and the sixth highest in the rTHA group. Prior studies have shown that increasing BMI is associated with increased LOS, costs, and intraoperative blood loss, which in turn can lead to a variety of postoperative complications [25].

Other comorbidities such as diabetes have been shown to affect postoperative outcomes. Lovecchio et al. elucidated the increased risk of medical complications in both insulin-dependent and non-insulin-dependent diabetics [26]. Insulin dependence was also described to be associated with higher readmission rates. Additionally, hypertension has a variable effect on cardiac complications. A systematic review by Elsiwy et al. discussed four independent articles evaluating the effect of hypertension on hip and knee joint arthroplasty outcomes, with two showing a positive correlation and two exhibiting no effect. The authors concluded that history of cardiac disease bore the strongest association with postoperative cardiac complications [27].

Optimization from these pre-existing comorbidities may lead to decreased length of stay duration, decreased post-discharge ED visits, and provide value by reducing cost burden to the health care system.

More recently, the implementation of advanced data analysis tools, such as machine learning algorithms, to better understand impact of a combination of various medical comorbidities on postoperative outcomes has been popularized [28–30]. Harris et al. demonstrated an accurate predictive model for mortality and complications following joint arthroplasty with patient-specific variables [31]. Such efforts would benefit from a better understanding of critical comorbidities, which could potentially be incorporated as predictive variables for these algorithms and would allow for the development of risk-stratification models with increasing accuracy. Once such models are available, and once postoperative outcomes can be predicted with relatively high accuracy based on comorbidity profiles, patient-specific payment models with a more distributed reimbursement system can be implemented. Ramkumar et al. utilized a preliminary Bayesian machine learning model trained with patient factors to forecast LOS and to quantify patient risk. The algorithm proposed a staggered payment model that reimburses based on patient risk level to reduce patient selection bias and promote access [32]. Such reimbursement models would diminish concerns for provider “cherry-picking” through fairly accounting for the heightened risk undertaken by surgeons performing THA and rTHA on a more complex population.

Similar to most large database cross-sectional observational studies, this study has several limitations. While the NIS supplies a large amount of healthcare and resource utilization data at the population level, it remains prone to frequent errors due to reliance on suboptimal coding systems and human manual entry of data [33]. Despite this inherent potential weakness, the database has been validated for complication and comorbidity data, and is considered an excellent tool to conduct population-based observational epidemiological studies. Additionally, the NIS is strictly limited to inpatient data, and hence NIS does not allow for a complete assessment of postoperative clinical and economic outcomes beyond the immediate in-hospital period. While this was not an initial aim for this study, a better understanding of the long-term postoperative course would further help in improving optimization efforts, and subsequent studies could focus on shedding further light onto that aspect of the episode of care. Furthermore, the NIS registry does not provide information such as operative technique, preoperative diagnostic information or intraoperative complications. This data would be helpful in further highlighting reasons behind complications following hip arthroplasty.

Despite its inherent limitations, the present study has considerable strengths in design and analysis. To the authors’ knowledge, this study constitutes the largest available comprehensive report at the population level delineating the notable medical comorbidities of THA and rTHA recipients. The substantially large volume of data and long duration of the study provide a generalizable cross-sectional understanding of the demographics, comorbidities, clinical and economic outcomes following THA and rTHA, in addition to the type and reason for rTHA. This knowledge can potentially empower clinicians with a deeper understanding of perioperative conditions that might impact postoperative outcomes and allow for development and implementation of perioperative optimization pathways geared to target these conditions.

## Conclusion

In conclusion, this study assessed the demographic and epidemiological characteristics of THA and rTHA recipients between 2006–2015 and reported significant medical comorbidities and postoperative outcomes associated with each cohort. In an era of value-based care delivery where focus is placed on achieving higher quality while simultaneously optimizing the efficiency of an episode of care, developing an understanding of the patient-specific comorbidities is paramount to designing personalized care protocols that subsequently improve outcomes. While some medical comorbidities prevalent among these cohorts have been extensively studied, this study identified gaps that will allow future studies to further assess additional comorbidities that were noted to be commonly encountered among THA and rTHA recipients.

## Data Availability

The datasets generated and/or analyzed during the current study are available in the National Inpatient Sample repository, https://www.hcup-us.ahrq.gov/db/nation/nis/nisdbdocumentation.jsp.

## Abbreviations

THA: Total Hip Arthroplasty
rTHA: Revision Total Hip Arthroplasty
LOS: Length of Stay
CPT: Current Procedural Terminology
